# Association between high immune activity and worse prognosis in uveal melanoma and low-grade glioma in TCGA transcriptomic data

**DOI:** 10.1186/s12864-022-08586-6

**Published:** 2022-05-07

**Authors:** Hitoshi Matsuo, Takashi Kamatani, Yu Hamba, Keith A. Boroevich, Tatsuhiko Tsunoda

**Affiliations:** 1 Laboratory for Medical Science Mathematics, Department of Biological Sciences, Graduate School of Science, The University of Tokyo, 7-3-1 Hongo, Bunkyo-ku, Tokyo, 113-0033 Japan; 2 Department of AI Technology Development, M&D Data Science Center, Tokyo Medical and Dental University, 2-3-10 Kandasurugadai, Chiyoda-ku, Tokyo, 113-8510 Japan; 3 Division of Precision Cancer Medicine, Tokyo Medical and Dental University Hospital, 2-3-10 Kandasurugadai, Chiyoda-ku, Tokyo, 101-0062 Japan; 4 Division of Pulmonary Medicine, Department of Medicine, Keio University School of Medicine, 160-8582 Tokyo, Japan; 5 Laboratory for Medical Science Mathematics, RIKEN Center for Integrative Medical Sciences, 230-0045 Yokohama, Japan; 6 Department of Computational Biology and Medical Sciences, Graduate School of Frontier Sciences, The University of Tokyo, 5-1-5 Kashiwanoha, Kashiwa, Chiba, 277-8562 Japan

**Keywords:** Endothelial mesenchymal transition, Glioma, Inflammation, Macrophages, Uveal melanoma

## Abstract

**Background:**

Immune status in the tumor microenvironment is an important determinant of cancer progression and patient prognosis. Although a higher immune activity is often associated with a better prognosis, this trend is not absolute and differs across cancer types. We aimed to give insights into why some cancers do not show better survival despite higher immunity by assessing the relationship between different biological factors, including cytotoxicity, and patient prognosis in various cancer types using RNA-seq data collected by The Cancer Genome Atlas.

**Results:**

Results showed that a higher immune activity was associated with worse overall survival in patients with uveal melanoma and low-grade glioma, which are cancers of immune-privileged sites. In these cancers, epithelial or endothelial mesenchymal transition and inflammatory state as well as immune activation had a notable negative correlation with patient survival. Further analysis using additional single-cell data of uveal melanoma and glioma revealed that epithelial or endothelial mesenchymal transition was mainly induced in retinal pigment cells or endothelial cells that comprise the blood-retinal and blood-brain barriers, which are unique structures of the eye and central nervous system, respectively. Inflammation was mainly promoted by macrophages, and their infiltration increased significantly in response to immune activation. Furthermore, we found the expression of inflammatory chemokines, particularly CCL5, was strongly correlated with immune activity and associated with poor survival, particularly in these cancers, suggesting that these inflammatory mediators are potential molecular targets for therapeutics.

**Conclusions:**

In uveal melanoma and low-grade glioma, inflammation from macrophages and epithelial or endothelial mesenchymal transition are particularly associated with a poor prognosis. This implies that they loosen the structures of the blood barrier and impair homeostasis and further recruit immune cells, which could result in a feedback loop of additional inflammatory effects leading to runaway conditions.

**Supplementary information:**

The online version contains supplementary material available at 10.1186/s12864-022-08586-6.

## Background

Immune status in the tumor microenvironment (TME) is important in determining cancer progression and patient prognosis. The effect of immune cell infiltration or immune activity level in the TME has received significant attention in the past decade. Cytotoxic T cells (CTLs) play an essential role in cancer elimination as they induce apoptosis in recognized cancer cells by releasing cytokines such as perforin and granzyme. Moreover, in tumors highly infiltrated with CTLs, cancer elimination occurs actively. In some tumors, CTL activity is inhibited because there is minimal CTL infiltration or cancer cells express checkpoint molecules and acquire systems to prevent attack by CTLs [1, 2]. Generally, tumors with a high cytotoxicity are correlated with a better patient prognosis [3], and therapies that activate tumoral immunity or enhance cytotoxicity have been developed [4–7].

However, some reports have shown that a high immune activity or immune cell infiltration is associated with a worse prognosis in certain cancer types [7, 8]. The abovementioned trend in cancer immunology is not common, and it may be affected by cancer type-specific factors. We focused on uveal melanoma (UVM) and glioma, which are cancers of the eye and central nervous system (CNS), respectively. These sites have a common anatomical structure, referred to as the blood-retinal barrier (BRB) in the eye and blood-brain barrier (BBB) in the CNS. The entry of immune cells into the tissues is regulated. Hence, both organs are referred to as immune-privileged sites and have immune systems different from other organs. We hypothesized that the prognostic impact of varying immune statuses could differ among them. Thus, the current study aimed to investigate how cytotoxicity and biological states and processes affect prognosis in UVM and glioma using transcriptomic data from The Cancer Genome Atlas (TCGA). Moreover, to assess poor prognostic factors specific to these cancer types, this correlation was compared across other cancer types. Subsequently, an analysis with additional single-cell data determined from which cells these factors originate, and the mechanisms associated with poor prognosis and histological features were identified.

## Results

### Association between high immune activity and poor overall survival in patients with UVM and LGG

**Fig. 1:**
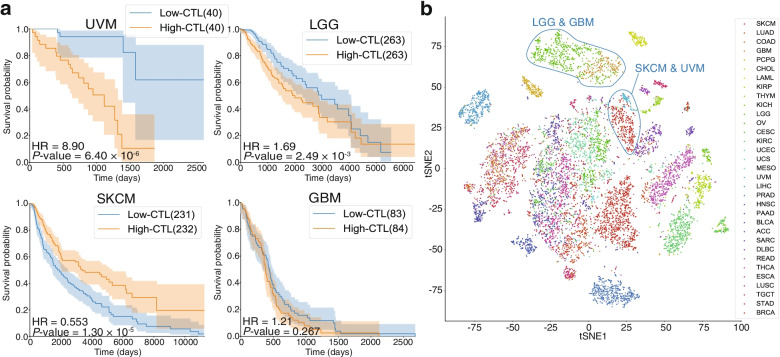
High immune activity was associated with poor overall survival in patients with UVM and LGG. **a** Kaplan-Meier curves of overall survival classified according to CTL levels (red: high CTL level, blue: low CTL level). The number samples are indicated in the legend. **b** tSNE plot based on the activity of well-defined biological states and processes calculated for all samples and color-coded according to cancer type. The names of the cancer types follow TCGA Study Abbreviations. EMT, epithelial mesenchymal transition

To evaluate immune activity status in the TME, we assessed the CTL levels in all TCGA samples and performed a survival analysis between the high and low CTL groups for each cancer type (Materials and Methods; Fig. S1a, b and c). In most cancers, including SKCM, the patient’s overall survival was significantly better in the high CTL group than in the low CTL group. However, in patients with UVM and low-grade glioma (LGG), the low CTL group had a significantly better overall survival than the high CTL group (Figs. 1a and S1c). Moreover, the survival did not differ between high and low CTL groups in patients with glioblastoma (GBM), which is a high-grade glioma. The CTL level was higher in patients with GBM than in those with LGG (Fig. S1a). To validate these findings, we performed the same analysis for gliomas in the CGGA dataset and found that a high CTL level was associated with a worse overall survival in LGG. Moreover, the immune activity of GBM was higher than that of LGG (Fig. S1b). The high CTL group had higher PD-L1 (CD274) expression than the low CTL group, which was common in all cancer types (Fig. S1d).

Next, we assessed well-known biological states and processes in the Hallmark gene sets registered in MSigDB by calculating the activities of these states and processes in all TCGA samples via ssGSEA. A dimensional compression analysis using tSNE showed clustering according to cancer type and organs with similar functions were plotted adjacent to each other (Fig. 1b). Interestingly, UVM and SKCM were almost in the same position (Fig. 1b). Melanocyte-derived cancers had similar biological states and processes compared with other types of cancers. The relationships between immune activity and prognosis were completely reversed between UVM and SKCM. Similarly, LGG and GBM clustered tightly with each other. Therefore, in the succeeding analysis, we focused on the differences between UVM and SKCM as well as LGG and GBM to assess the prognostic factors of UVM and LGG.

### Hazard ratios of inflammation and EMT in UVM and LGG

**Fig. 2:**
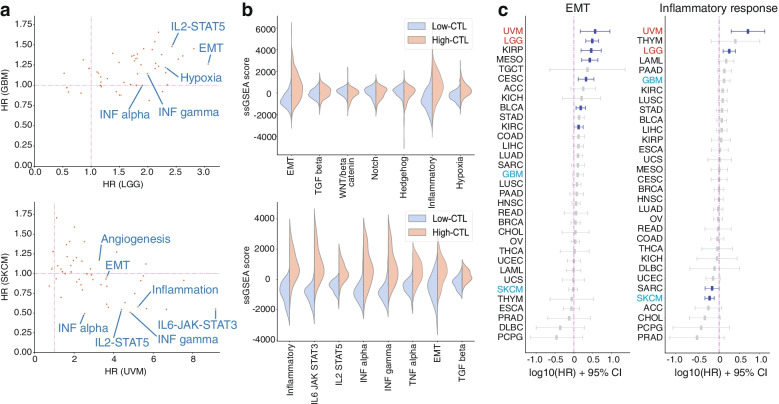
Hazard ratios of inflammation and EMT in UVM and LGG. **a** Hazard ratio of each state and process in LGG and GBM (top) and in UVM and SKCM (bottom). **b** Difference in activity score for some states and processes between the high and low CTL groups in LGG (top) and UVM (bottom). **c** Logarithm of hazard ratios for EMT (left) and inflammatory response (right) among different cancer types. Those with a *p-*value of < 0.05 are depicted in blue and others in gray. The names of the cancer types follow TCGA Study Abbreviations. EMT, epithelial mesenchymal transition

To examine which biological states and processes in ssGSEA that were strongly associated with worse overall survival in UVM and LGG, we calculated their hazard ratios (HRs). Results showed that epithelial mesenchymal transition (EMT) had the highest HR for worse survival in LGG (Fig. 2a). On the other hand, the HR of EMT in GBM was close to 1 (Fig. 2a; Table S1). In LGG, the activity score of EMT was positively correlated with the CTL level and was significantly enhanced in the high CTL group compared with the low CTL group (Figs. 2b and S2c; Table S2). Moreover, these results were confirmed via an analysis using the CGGA dataset (Fig. S2a and b). To investigate the cause of the enhanced EMT and immune activation, we focused on the upstream signaling pathways associated with the former. In gliomas, EMT is promoted by the TGF-beta, Wnt-beta catenin, Notch, and Hedgehog signaling pathways [9–11]. The TGF-beta pathway alone was found to be minimally activated in the high CTL group, as assessed using the TCGA dataset (Fig. 2b). However, this result could not be validated using the CGGA dataset (Fig. S2b). The activity of the Notch and Hedgehog signaling pathways was not correlated with the differences in immune activation status, and there was greater Wnt-beta activation in the low CTL group than in the high CTL group (Figs. 2b, S2b, c and d). By contrast, inflammation and hypoxia are also known to enhance EMT in several brain diseases [9, 11], and these scores were significantly increased in the high CTL group in our results (Fig. 2b). The activities of the inflammatory signaling pathways, such as the IL2-STAT5, IL6-STAT3, interferon-alpha (INF-α), interferon-gamma (INF-γ), and tumor necrosis factor alpha (TNF-α), were positively correlated with the CTL level and were significantly more activated in the high CTL group compared to the low CTL group (Figs. 2b, S2b, c and d; Table S2). Furthermore, the HRs of these pathways were higher in LGG than in GBM (Fig. S2e).

Next, we compared the HRs of each biological state/process for worse overall survival between UVM and SKCM. The IL6-JAK-STAT3, IL2-STAT5, INF-α/γ, and TNF-α pathways had significantly higher and lower HRs in UVM and SKCM, respectively (Fig. 2a; Table S3). The activities of these signaling pathways were positively correlated with CTL levels and were significantly activated in the high CTL group (Figs. 2b and S3a; Table S4). The HR of the EMT in LGG was significantly higher in UVM, and it was close to 1 in SKCM (Fig. 2a). In some eye diseases, EMT is enhanced after inflammation or the activation of the TGF-beta pathway [12]. Our results show that inflammation was enhanced, as described above, and the TGF-beta pathway was significantly activated in the high CTL group (Figs. 2b, S3a). Among all of the cancer types in TCGA, UVM and LGG had the two highest HRs of EMT, inflammatory response and hypoxia for worse survival (Figs. 2c and S3b).

### Types of cells causing inflammatory effects and EMT in UVM and LGG

**Fig. 3:**
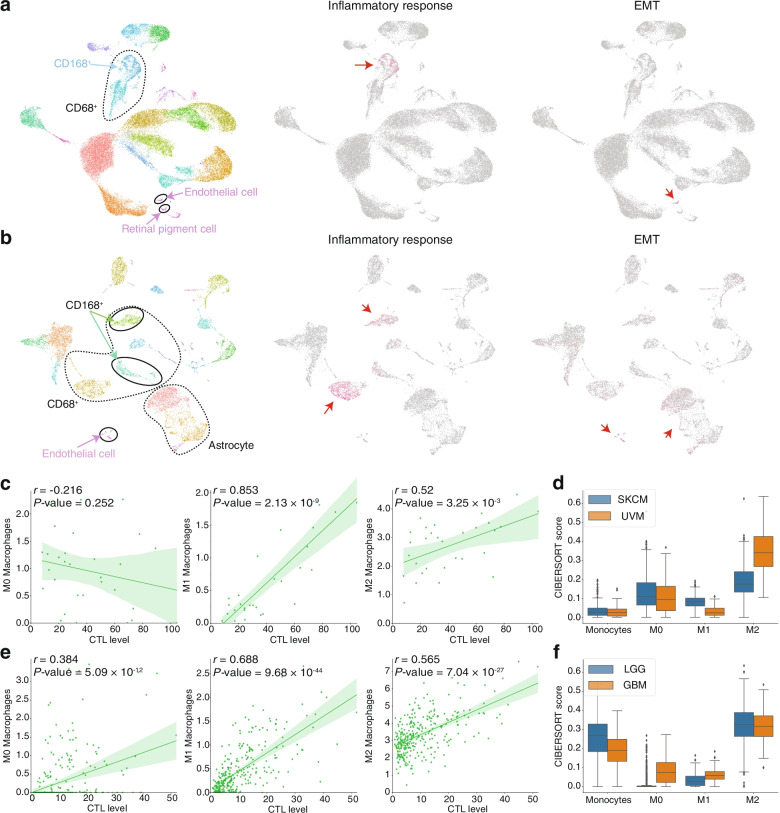
Single-cell analysis and the macrophage relative abundance level in UVM and LGG. **a, b** Umap projection of clustering analysis (left), and the overview of activation scores of inflammation (middle) and EMT (right) across all cells in UVM (**a**) and LGG (**b**). **c, d** Correlation between M0, M1, and M2 macrophage levels determined using CIBERSORT (absolute = T) and CTL levels in UVM (**c**) and LGG (**d**). **e, f** The fraction of monocyte and M0, M1, and M2 macrophages in the tumor determined using CIBERSORT (absolute = F) in UVM and SKCM (**e**) and LGG and GBM (**f**). SKCM, skin cutaneous melanoma; UVM, uveal melanoma; LGG, low-grade glioma; GBM, glioblastoma

As presented in the previous section, inflammatory effects and EMT were found to be strongly correlated with worse prognosis in UVM and LGG. To determine which cells are responsible for the inflammatory effects and EMT, we used single-cell RNA-seq data from GEO (GSE139829 and GSE138794) [13, 14]. We calculated the activity score of IL6-STAT3, IL2-STAT5, INF-α/γ, TNF-α, and inflammatory states via ssGSEA using the single-cell data of eight primary UVM samples. The Umap analysis revealed several cell clusters with high inflammatory effects (Figs. 3a and S4b). In these clusters, CD68 and CD163 are expressed, both of which are known markers of macrophages (Figs. 3a, S4a and b). NFkB (*NFKB1*) and COX-2 (*PTGS2*), known as inflammation-related molecules associated with a bad prognosis of Uveal melanoma [15], were highly expressed mainly in the macrophage population. Also, consistent with the previous study’s finding, CXCL10, known as inflammation-inducing and lymphocyte-attracting chemokines [16], was highly expressed exclusively in the same population (Fig. S4c). The HR of angiogenesis was the highest in UVM compared to other cancer types and was also significantly enhanced in the high CTL group (Table S2). Next, cell populations with EMT had a high expression of endothelial and retinal pigmented epithelial cell markers (Figs. 3a, S4a and b). From the single-cell data of nine glioma samples, we identified inflammatory cell populations that were positive for CD68 (Fig. 3b, S4d and e). In contrast to UVM, CD163 positive cells showed only a slight inflammatory response in glioma (Figs. 3b, S4d and e). The cell populations in which EMT was activated were mainly cells expressing endothelial cell markers, similar to what was seen in UVM (Figs. 3b, S4d and e). Because CD163 indicates M2-like macrophages, the inflammatory effect, which was associated with worse prognosis in UVM and LGG, is mainly observed in macrophage populations that differ in phenotype from each other: M2-like macrophages in UVM and M0/M1-like macrophages in LGG.

Next, to validate whether tumors with a higher immune activity have an increased abundance of macrophages, we evaluated the immune status of the tumors using CIBERSORT [17]. The absolute abundance scores of M1 and M2 macrophages in UVM were positively correlated with the CTL level. A similar correlation was observed in the xCell scores [18], indicating that macrophages were relatively more abundant in the state of high immune activity (Figs. 3c and S5a). The ratios of each immune cell showed that M2 macrophages were the most predominant cells in UVM (Fig. S5c). The percentages of macrophages and monocytes in the tumor were compared between UVM and SKCM. Results showed that M2 macrophages were more abundant in UVM than in SKCM (Fig. 3d).

In LGG, the proportion of M0, M1, and M2 macrophages was significantly increased in tumors with higher CTL levels, and a similar correlation was observed in the xCell scores (Figs. 3e and S5b). Furthermore, a survival analysis was performed by dividing the abundance scores of M1 and M2 macrophages by the median. Results showed that worse survival was significantly associated with a high macrophage invasion (Fig. S5c). Because the M0 macrophage score was 0 in more than half of the samples, survival analysis could not be performed for M0. In addition, we compared the percentage of macrophages (combined monocyte and M0, M1, and M2 macrophages) in all cancer types. In LGG and GBM, the macrophages were significantly abundant, and glioma was the macrophage-dominant cancer (Fig. S5f). By comparing the percentages of macrophages in LGG and GBM, the fraction of M0 and M1 macrophages increased in GBM (Fig. 3f). Results showed that increased relative abundance of macrophages was associated well with immune activation.

### High correlation between the expression of inflammatory mediator chemokine CCL5 and CTL level

**Fig. 4:**
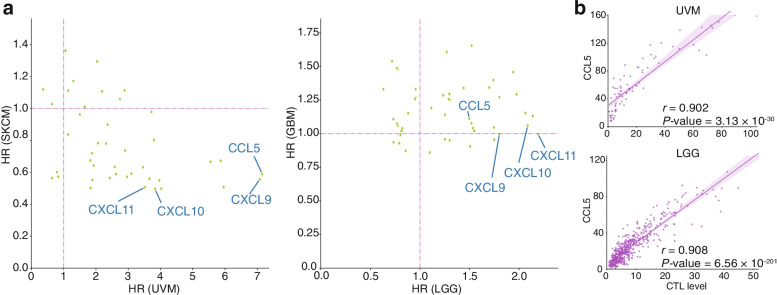
Association between chemokine expression as well as overall survival and CTL levels. **a** Hazard ratio of each chemokine in UVM and SKCM (left) and LGG and GBM (right). **b** Correlation between CCL5 expression and CTL levels in UVM (top) and LGG (bottom). SKCM, skin cutaneous melanoma; UVM, uveal melanoma; LGG, low-grade glioma; GBM, glioblastoma

We investigated potential molecules that can improve the function of BRB and BBB and inhibit the infiltration of immune cells, such as macrophages. Here we focused on chemokines because they are a class of cytokines involved in the development of inflammation by promoting the migration of leukocytes and other immune cells, and they have recently attracted attention as targets for cancer therapeutics. We calculated the HR of each chemokine for worse prognosis based on expression level. Results showed that *CCL5* had the highest HR in UVM (Fig. 4a; Table S5). CCL5 is known to promote BBB disruption, and the HR of *CCL5* was significantly high in LGG, whereas it was low in SKCM and close to 1 in GBM. The expression of *CCL5* was most positively correlated with CTL level in LGG, and a high correlation was also observed in UVM (Fig. 4b; Table S6). CXCL11, which is a ligand of CXCR3, had the highest HR in LGG. CXCR3 has ligands including CXCL9, CXCL10, and CXCL11. These chemokines had high HRs in both UVM and LGG, and they were also strongly correlated with CTL levels (Tables S5, S6 and S7).

## Discussion

Contrary to the general trend of tumor immunity, the prognosis was significantly worse in the high CTL group than in the low CTL group in UVM and LGG. On the other hand, in SKCM, to which checkpoint inhibitor was first applied, the prognosis was significantly better in the high CTL group. A tSNE plot generated based on the activity scores of well-defined biological states and processes as determined via ssGSEA showed that UVM and SKCM, both of which are derived from melanocytes, resided in almost the same cluster, indicating that they share common biological properties. Therefore, high immune activity being associated with a poor prognosis in UVM is likely due to the tissue-specific histological nature of the eye rather than the molecular endogenous characteristics of each cell within the tumor. The BRB and BBB are the common structures of the eye and CNS; these are physical barriers created by tight junctions around vascular endothelial cells that line the space between capillaries and tissues and various cells surrounding them (such as optic nerve cells in the eye or pericytes and astrocytes in the CNS). The outer BRB is comprised of pigmented retinal epithelial cells, and the inner BRB of capillary endothelial cells [19]. We considered that this histological feature is one of the factors of the poor prognosis in cases of high cytotoxicity, as discussed below.

Inflammatory effects and EMT were strong poor prognostic indicators of UVM and LGG. Furthermore, most inflammatory effects were seen in macrophage populations, and EMT was observed in epithelial and endothelial cell populations. Endothelial mesenchymal transition (EndoMT) is almost the same conversion mechanism as EMT induced in endothelial cells, suggesting that EMT from ssGSEA found in endothelial cells is a proxy for EndoMT. In some eye and brain diseases, the epithelial and endothelial cells comprising the BRB and BBB are known to cause EMT and EndoMT, respectively [9, 12]. Therefore, the same mechanism can occur in UVM and LGG. In particular, EMT was most associated with a worse prognosis in LGG. In the brain, the TGF-beta, Wnt-beta catenin, NOTCH, and Hedgehog signal pathways activate EMT and EndoMT [9–11]. However, there were not sufficient results to show that these pathways were activated in the high CTL group. On the other hand, inflammatory state and hypoxia, which were significantly enhanced in the high-CTL group, are also known to promote EMT/EndoMT in the brain. These states increase the permeability of the BBB and make endothelial cells more prone to exhibit EndoMT and also loosen the tight junctions of the BBB [9, 11]. The current study suggests that with immune activation, macrophage abundance increased, and the inflammatory effects from macrophages also became stronger. Furthermore, compared with other cancer types, inflammation response and hypoxia were strongly associated with worse prognosis in LGG, indicating that inflammation and hypoxia may strongly contribute to the induction of EMT/EndoMT. The BBB essentially prevents immune cell infiltration [20, 21], which suggests that this histological feature is a factor in the poor prognosis observed in cases of high cytotoxicity. When this structure undergoes EndoMT, the tight junctions loosen and vascular permeability increases, thereby allowing the infiltration of large immune cells, such as macrophages, which normally cannot pass [9]. Gliomas are macrophage-dominant cancers, and they are correlated with an abundance of microglia, a type of glial cell, and resident macrophages, in the CNS. Previous studies have shown that in low-grade glioma, microglia are distributed closer to the center of tumor and circulating macrophages derived from monocytes invade the tumor beyond the BBB [22]. These findings suggest a positive feedback loop in which macrophages infiltrate a tumor and enhance inflammation, promoting EMT/EndoMT to the BBB, which in turn recruits additional inflammatory macrophages. In addition, EMT was not associated with a worse prognosis in GBM. In advanced gliomas, including GBM, the BBB is often disrupted due to tumor overgrowth. This is consistent with the hypothesis that EMT occurs in the cells comprising the BBB.

In UVM, the current study showed that inflammatory pathways such as the TNF-α, as well as EMT, were strongly activated in the high CTL group, indicating a possibility that inflammation had a significant influence on the induction of EMT/EndoMT. This is consistent with the previous studies reporting that oxidative stress causes dissociations of cell-cell adhesion of retinal pigment epithelial cells, which constitute the BRB, and TNF-α induces the EMT of retinal pigmented epithelial cells [23, 24]. Our data also suggested that the source of the inflammatory effect is likely to be macrophages and showed an increased relative abundance of macrophages in line with immune activation. Macrophages have a substantial effect on angiogenesis [25], and our results also showed a strong correlation between poor prognosis and angiogenesis. Angiogenesis was positively associated with immune activation, suggesting that further immune cell infiltration can be induced by chemokines and other molecules released from macrophages. Regarding the BRB, since UVM is mainly developed in the choroid, it is unclear whether the failure of the BRB is responsible for the increased infiltration of immune cells in the tumor, as in the case of LGG. However, the BRB is a critical structure that maintains ocular homeostasis [26]. Its disruption may contribute to the instability of the ocular environment. These findings suggest a feedback model starting from macrophages’ inflammatory effect along with promoting angiogenesis to further infiltration of immune cells, as well as an additional feedback loop of EMT to BRB structure, similar to LGG, which disrupts the ocular homeostasis.

Furthermore, our results show M2 macrophages are abundant in UVM, while the proportion of M0 and M1 macrophages increased in GBM compared with LGG. This is consistent with the single-cell analysis data showing that inflammatory effects were observed in CD163 positive (M2-like) macrophages in UVM and CD163 negative (M0 or M1-like) macrophages in glioma. Hence, the inflammatory effect of M1-like macrophages may have a negative impact on prognosis in the CNS. In a mouse glioma model, the inflammatory effect of M1 macrophages under hypoxic conditions can damage even normal neuron cells [27]. Moreover, there are autoimmune diseases of the uvea that cause severe vision loss due to the inflammatory effects of resident immune cells [28]. Considering that the eye and CNS are immune-privileged sites, that resist immunogenic inflammation through multiple mechanisms, these organs may be natively sensitive to inflammation.

Considering the possibility that the inflammatory effects from macrophages may trigger a feedback loop through loosening the structure or inducing angiogenesis and cause further macrophages infiltration, we suggest that repair of these structures and suppression of inflammation may be able to improve prognosis. To investigate this, we focused on chemokines. In recent years, checkpoint inhibitors have been developed to improve treatment modalities by regulating the expression or inhibition of specific interleukins and chemokines [29]. Our results showed that the expression level of the chemokine *CCL5* correlated most strongly with immune activity and poor prognosis in both LGG and UVM. CCL5 has roles as an inflammatory mediator and in the structural disruption of the BBB, and inhibition of CCL5 can repair the disrupted BBB [29]. It has been reported that symptoms are ameliorated in a CCL5 knockout mouse model of cerebral infarction [30]. Moreover, microglias respond to CCL5 and promote the disruption of BBB in later stages via inflammatory effects [31]. Similarly, expression levels of the CXCR3 ligands CXCL9/10/11 were strongly correlated with CTL levels and were strongly associated with worse prognosis. Although detailed functions of these ligands are not yet well understood, they have been, CXCL10 in particular, reported being involved in suppressing or promoting inflammation in the nervous system [32, 33]. In UVM, our results showed that CXCL10 is expressed mainly in macrophages. A previous mouse study showed that depletion of macrophages suppressed UVM tumor growth [34]. These observations suggest that treatment strategies targeting these chemokines to control inflammation while maintaining normal tissue structure may lead to better treatment.

This study has limitations. It is difficult to clearly identify the causality between inflammation and cancer progression, or the primary source of inflammation. Our single-cell analysis suggested that macrophages may have a stronger inflammatory effect, but as inflammation may also be caused by the cancer cells themselves, it is difficult to determine which is a more robust inflammatory source. It is possible that inflammation may just appear stronger because of the progression of cancer. Because the sample size of single cells is also small (less than 10 samples), more samples are needed in the future to investigate and strengthen the validation. In addition, because likely prognostic factors were identified from pathways registered in MSigDB as HALLMARK, additional factors other than EMT and inflammation cannot be ruled out. The hypothesis that inflammatory effects cause damage to the blood barrier was also interpreted on the basis of previous studies. Depending on the origin of the inflammation, there may be some mediator between the inflammatory action and the disruption of the blood barrier. Future work is needed to confirm these, such as experimentally in a mouse model of glioma or Uveal melanoma along a time course.

## Conclusions

A high tumoral immune activity does not necessarily indicate a better prognosis, and the histological specificity of each organ may have a significant role. In UVM and LGG, the prognosis is better with low immune activity, even though this is contrary to the general concept of tumor immunity. Our data also shows promoted inflammation response originated from macrophages and increased expression of inflammatory chemokine, such as CCL5 and CXCL10, along with immune activation. These effects are known to cause further EMT/EndoMT and loose the blood barrier structure, which maintains environmental homeostasis by tightly controlling the interaction between blood vessels and tissue components in the eye and CNS. Our result suggests that when immunity is activated, EMT and EndoMT are induced in cells comprising the blood barrier, which is highly involved in poor prognosis in these cancers. Further recruitment of immune cells may be led by the disruption of this structure in LGG and by increased angiogenesis promoted by macrophages in UVM. Therefore, we suggest that this mechanism may result in additional inflammatory effects through a deleterious positive feedback loop. Considering the histological impact of each cancer type, reducing excessive immune cell infiltration, maintaining the tissue structure and controlling inflammation status could be a part of an effective immunotherapy strategy.

## Materials and methods

### Data preparation

We used data available from The Cancer Genome Atlas (TCGA) project [35]. We downloaded the mRNA sequencing results and clinical information of 11,057 samples from 33 different cancer types from the UCSC Xena (http://xenabroser.net/datapages/). The mRNA data were provided in FPKM, and the total number of reads for each sample was disparate. Therefore, the expression of each gene was transformed by multiplying by 1,000,000/total number of reads. For validation, we used RNA-seq and clinical data of the Chinese Glioma Genome Atlas (CGGA), which is a public genomic database of Chinese patients with glioma [36, 37]. The mRNA sequencing data and clinical information including overall survival for prognosis of 693 patients with glioma were downloaded. Gene expressions were provided as RSEM, and no specific correction was made.

We obtained single-cell RNA-seq data from eight primary UVM samples (GSE139829) [13] and nine glioma samples (GSE138794) [14]. The data were combined per cancer type using the Seurat package (4.0.0) in R (4.0.3). Filtering was conducted based on previous studies [13, 14]. For GSE139829 data, cells were retained that had unique molecular identifiers (UMIs) greater than 400, between 100 and 8000 expressed genes (inclusive), and less than 10% mitochondrial content. For GSE138794 data, retained cells had UMIs greater than 200, expressed more than 1 gene, and had mitochondrial content less than 5%. Sample batch correction was not performed. Data were normalized using the LogNormalize method, and the scale factor was 10,000. Visualization was conducted using Umap. As in previous studies [13, 14], we used markers for immune cells: CD14, CD68, and CD163 for macrophages; CLDN5 and VWF for endothelial cells; RPE65 for retinal pigment cells (GSE139829 only); MITF for cancer cells (GSE139829 only); and GFPA and AQP4 for astrocytes (GSE138794 only).

### Immune scores, biological states and process activity in the tumor microenvironment

The CTL level was used to indicate tumoral immune activity. The sum of the expression values of five genes (*CD8A*, *CD8B*, *GZMA*, *GZMB*, and *PRF1*) was used to calculate the CTL level according to previous studies [38, 39]. High and low CTL groups were defined relatively using the median value of CTL levels within the group.

We performed a single sample gene set enrichment analysis (ssGSEA) to determine the activity level of each signaling pathway, states, or processes in the gene expression profile. The GSVA package (1.34.0) in R (3.6.3) was used for bulk RNA-seq data analysis with the following parameters: method = ssgsea, tau = 0.75, ssgsea.norm = F. Data were zero-mean normalized [40]. The script was implemented with reference to the code of Immunoduct (https://github.com/msfuji/immunoduct/tree/master/scripts). For single-cell RNA-seq samples, the escape package (1.1.1) in R (4.0.3) was utilized to perform ssGSEA [41]. Moreover, the Hallmark gene sets, which are registered in the Molecular Signatures Database (MSigDB) version 7.4, were applied [42].

We ran cell-type identification by estimating the relative subsets of RNA transcripts **(**CIBERSORT) to estimate the relative abundances (“absolute mode” of CIBOERSORT) of various immune cells in the TME based on the mRNA expression profile [17]. We used LM22 as the signature gene file, and permutation was set to 100. The command line script for CIBERSORT version 1.04 was used in R (3.6.3), and only results with a *p*-value of < 0.05 were utilized. When determining the percentage of each immune cell in the tumor, absolute mode was set to false. The script was implemented with reference to the code of Immunoduct. In addition, we used another signature-based method to infer immune cells (xCell). xCell data from the TCGA are available on the website [18], and they were used to validate CIBERSORT results.

### Statistical analysis

Survival and statistical analyses were conducted using the lifelines package (0.25.9) and the scipy package (1.6.1) in Python (3.8.5). Kaplan-Meier curves were used to assess the influence of different factors on patient overall survival, and the log-rank test was utilized to calculate the *p*-value. The patients were divided into two groups using the median of each calculated score (such as the CTL level and CIBERSORT scores), and hazard ratios for patient overall survival were calculated utilizing the Cox proportional hazard regression model, with samples below the median as 0 and those above the median as 1. Data between the two groups were compared using the Welch’s *t*-test, and the Pearson’s correlation coefficient was used to evaluate correlations. A *p*-value of < 0.05 was considered statistically significant. Bonferroni’s correction was applied for multiple testing.

## Supplementary Information

**Additional file 1.**

**Additional file 2.**

## Data Availability

All data used in this study are included in these published articles [13, 14, 35–37].

## Abbreviations

BBB: blood-brain barrier
BRB: blood-retinal barrier
CNS: central nervous system
CTL: cytotoxic T lymphocyte
EndoMT: endothelial mesenchymal transition
EMT: epithelial mesenchymal transition
GBM: glioblastoma
HR: hazard ratio
LGG: low-grade glioma
SKCM: skin cutaneous melanoma
TME: tumor microenvironment
UVM: uveal melanoma
