# Supplementary material for: Association between high immune activity and worse prognosis in uveal melanoma and low-grade glioma in TCGA transcriptomic data
Source: BMC Genomics. 2022 May 7;23:351. doi: 10.1186/s12864-022-08586-6 (PMC9078026; doi:10.1186/s12864-022-08586-6)
Supplement: Supplementary file 1 — Additional file 1. [file 12864_2022_8586_MOESM1_ESM.docx]

**Table S1** Hazard ratios and their *p*-values for well-defined biological states and processes in LGG and GBM sorted by HR(LGG).

**Table S2** Results of Welch's t-test for differences in the activity of each state or process between High-CTL and Low-CTL groups on LGG and GBM sorted by *t*-Statistic(LGG).

**Table S3** Hazard ratios and their p-values for well-defined biological states and processes in UVM and SKCM sorted by HR(UVM).

**Table S4** Results of Welch's t-test for differences in the activity of each state or process between High-CTL and Low-CTL groups on UVM and SKCM sorted by *t*-Statistic(UVM).

**Table S5** Hazard ratios and their *p*-values for chemokines in UVM and SKCM sorted by HR(UVM).

**Table S6** Hazard ratios and their *p*-values for chemokines in LGG and GBM sorted by HR(LGG).

**Table S7** Correlations of chemokines expression and the CTL level in UVM and LGG sorted by Pearson’s *r*(LGG).
