# Supplementary material for: Association between high immune activity and worse prognosis in uveal melanoma and low-grade glioma in TCGA transcriptomic data
Source: BMC Genomics. 2022 May 7;23:351. doi: 10.1186/s12864-022-08586-6 (PMC9078026; doi:10.1186/s12864-022-08586-6)
Supplement: Supplementary file 2 — Additional file 2. [file 12864_2022_8586_MOESM2_ESM.pdf]

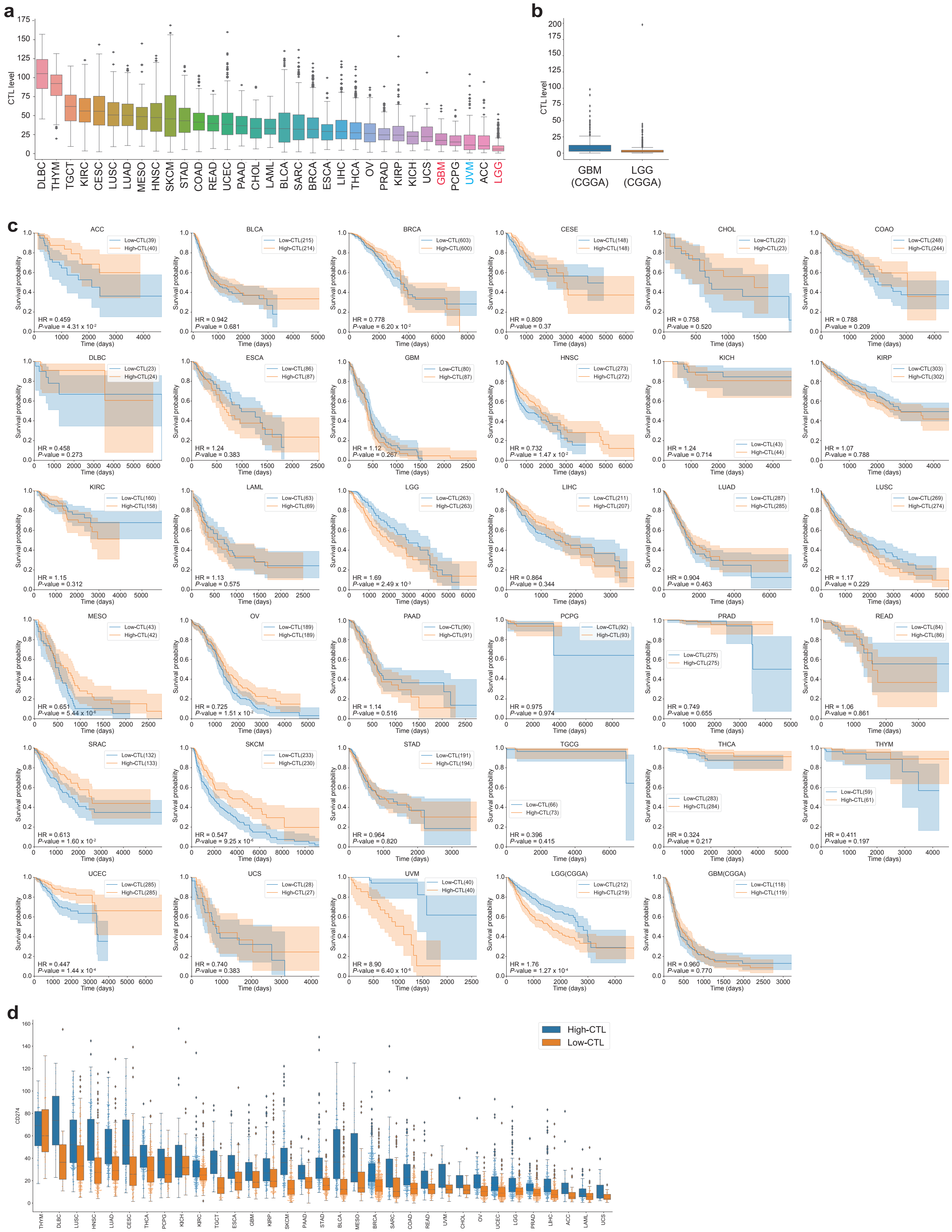
a
CTL level
DLBC THYM TGCT KIRC CESC LUSC LUAD MESO HNSC SKCM STAD COAD READ UCEC PAAD CHOL LAML BLCA SARC BRCA ESCA LIHC THCA OV PRAD KIRP KICH UCS GBM PCPG UVM ACC LGG
b
CTL level
GBM (CGGA)
LGG (CGGA)
c
Survival probability
Time (days)
ACC Low-CTL(39) High-CTL(40) HR = 0.459 P-value = 4.31 x 10-2
BLCA Low-CTL(215) High-CTL(214) HR = 0.942 P-value = 0.681
BRCA Low-CTL(603) High-CTL(600) HR = 0.778 P-value = 6.20 x 10-2
CESE Low-CTL(148) High-CTL(148) HR = 0.809 P-value = 0.37
CHOL Low-CTL(22) High-CTL(23) HR = 0.758 P-value = 0.520
COAO Low-CTL(248) High-CTL(244) HR = 0.788 P-value = 0.209
DLBC Low-CTL(23) High-CTL(24) HR = 0.458 P-value = 0.273
ESCA Low-CTL(86) High-CTL(87) HR = 1.24 P-value = 0.383
GBM Low-CTL(80) High-CTL(87) HR = 1.12 P-value = 0.267
HNSC Low-CTL(273) High-CTL(272) HR = 0.732 P-value = 1.47 x 10-2
KICH Low-CTL(43) High-CTL(44) HR = 1.24 P-value = 0.714
KIRP Low-CTL(303) High-CTL(302) HR = 1.07 P-value = 0.788
KIRC Low-CTL(160) High-CTL(158) HR = 1.15 P-value = 0.312
LAML Low-CTL(63) High-CTL(69) HR = 1.13 P-value = 0.575
LGG Low-CTL(263) High-CTL(263) HR = 1.69 P-value = 2.49 x 10-3
LIHC Low-CTL(211) High-CTL(207) HR = 0.864 P-value = 0.344
LUAD Low-CTL(287) High-CTL(285) HR = 0.904 P-value = 0.463
LUSC Low-CTL(269) High-CTL(274) HR = 1.17 P-value = 0.229
MESO Low-CTL(43) High-CTL(42) HR = 0.651 P-value = 5.44 x 10-3
OV Low-CTL(189) High-CTL(189) HR = 0.725 P-value = 1.51 x 10-2
PAAD Low-CTL(90) High-CTL(91) HR = 1.14 P-value = 0.516
PCPG Low-CTL(92) High-CTL(93) HR = 0.975 P-value = 0.974
PRAD Low-CTL(275) High-CTL(275) HR = 0.749 P-value = 0.655
READ Low-CTL(84) High-CTL(86) HR = 1.06 P-value = 0.861
SRAC Low-CTL(132) High-CTL(133) HR = 0.613 P-value = 1.60 x 10-2
SKCM Low-CTL(233) High-CTL(230) HR = 0.547 P-value = 9.25 x 10-6
STAD Low-CTL(191) High-CTL(194) HR = 0.964 P-value = 0.820
TGCG Low-CTL(66) High-CTL(73) HR = 0.396 P-value = 0.415
THCA Low-CTL(283) High-CTL(284) HR = 0.324 P-value = 0.217
THYM Low-CTL(59) High-CTL(61) HR = 0.411 P-value = 0.197
UCEC Low-CTL(285) High-CTL(285) HR = 0.447 P-value = 1.44 x 10-4
UCS Low-CTL(28) High-CTL(27) HR = 0.740 P-value = 0.383
UVM Low-CTL(40) High-CTL(40) HR = 8.90 P-value = 6.40 x 10-6
LGG(CGGA) Low-CTL(212) High-CTL(219) HR = 1.76 P-value = 1.27 x 10-4
GBM(CGGA) Low-CTL(118) High-CTL(119) HR = 0.960 P-value = 0.770
d
High-CTL
Low-CTL
CD274
THYM DLBC LUSC HNSC LUAD CESC THCA PCPG KICH KIRC TGCT ESCA GBM KIRP SKCM PAAD STAD BLCA MESO BRCA SARC COAD READ UVM CHOL OV UCEC LGG PRAD LIHC ACC LAML UCS

Figure S1 a The expression level of CTL by cancer type in TCGA. **b** The expression level of CTL of LGG and GBM using CGGA dataset. **c** Kaplan-Meier curves of overall survival split by the CTL level across cancer type in TCGA and in CGGA. **d** Difference in PD-L1 (CD274) expression between High-CTL and Low-CTL groups across cancer type in TCGA.

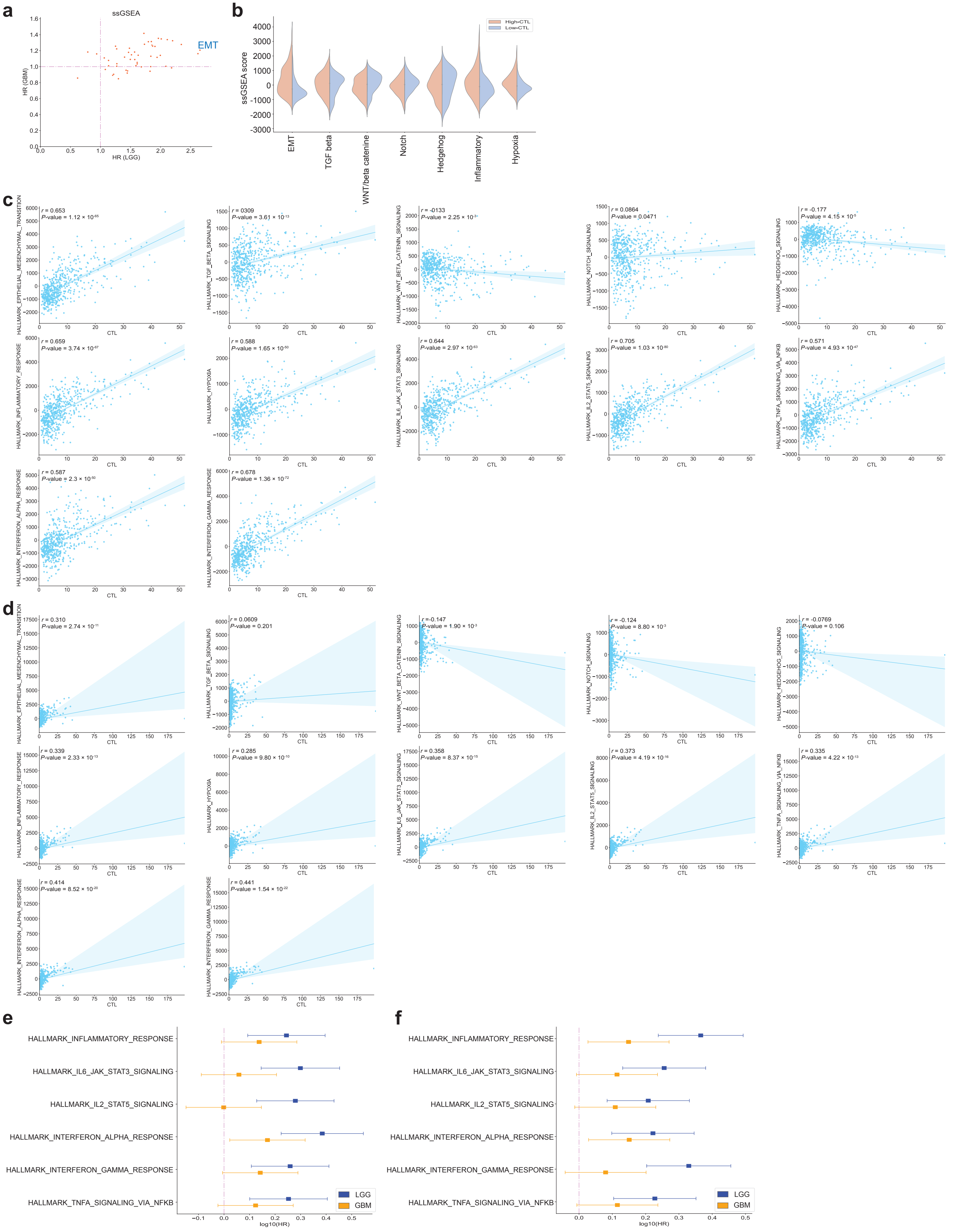

a
ssGSEA
EMT
HR (GBM)
HR (LGG)
b
ssGSEA score
High-CTL
Low-CTL
EMT
TGF beta
WNT/beta catenine
Notch
Hedgehog
Inflammatory
Hypoxia
c
HALLMARK_EPITHELIAL_MESENCHYMAL_TRANSITION
r = 0.653
P-value = 1.12 × 10^-65
HALLMARK_TGF_BETA_SIGNALING
r = 0309
P-value = 3.61 × 10^-13
HALLMARK_WNT_BETA_CATENIN_SIGNALING
r = -0133
P-value = 2.25 × 10^-3
HALLMARK_NOTCH_SIGNALING
r = 0.0864
P-value = 0.0471
HALLMARK_HEDGEHOG_SIGNALING
r = -0.177
P-value = 4.15 × 10^-5
HALLMARK_INFLAMMATORY_RESPONSE
r = 0.659
P-value = 3.74 × 10^-67
HALLMARK_HYPOXIA
r = 0.588
P-value = 1.65 × 10^-50
HALLMARK_IL6_JAK_STAT3_SIGNALING
r = 0.644
P-value = 2.97 × 10^-63
HALLMARK_IL2_STAT5_SIGNALING
r = 0.705
P-value = 1.03 × 10^-80
HALLMARK_TNFA_SIGNALING_VIA_NFKB
r = 0.571
P-value = 4.93 × 10^-47
HALLMARK_INTERFERON_ALPHA_RESPONSE
r = 0.587
P-value = 2.3 × 10^-50
HALLMARK_INTERFERON_GAMMA_RESPONSE
r = 0.678
P-value = 1.36 × 10^-72
CTL
d
HALLMARK_EPITHELIAL_MESENCHYMAL_TRANSITION
r = 0.310
P-value = 2.74 × 10^-11
HALLMARK_TGF_BETA_SIGNALING
r = 0.0609
P-value = 0.201
HALLMARK_WNT_BETA_CATENIN_SIGNALING
r =-0.147
P-value = 1.90 × 10^-3
HALLMARK_NOTCH_SIGNALING
r = -0.124
P-value = 8.80 × 10^-3
HALLMARK_HEDGEHOG_SIGNALING
r = -0.0769
P-value = 0.106
HALLMARK_INFLAMMATORY_RESPONSE
r = 0.339
P-value = 2.33 × 10^-13
HALLMARK_HYPOXIA
r = 0.285
P-value = 9.80 × 10^-10
HALLMARK_IL6_JAK_STAT3_SIGNALING
r = 0.358
P-value = 8.37 × 10^-15
HALLMARK_IL2_STAT5_SIGNALING
r = 0.373
P-value = 4.19 × 10^-16
HALLMARK_TNFA_SIGNALING_VIA_NFKB
r = 0.335
P-value = 4.22 × 10^-13
HALLMARK_INTERFERON_ALPHA_RESPONSE
r = 0.414
P-value = 8.52 × 10^-20
HALLMARK_INTERFERON_GAMMA_RESPONSE
r = 0.441
P-value = 1.54 × 10^-22
CTL
e
f
HALLMARK_INFLAMMATORY_RESPONSE
HALLMARK_IL6_JAK_STAT3_SIGNALING
HALLMARK_IL2_STAT5_SIGNALING
HALLMARK_INTERFERON_ALPHA_RESPONSE
HALLMARK_INTERFERON_GAMMA_RESPONSE
HALLMARK_TNFA_SIGNALING_VIA_NFKB
LGG
GBM
log10(HR)

Figure S2 a Hazard ratio for each state or process in LGG and GBM using CGGA dataset. **b** Difference in activity score for some states or process between High-CTL and Low-CTL groups in LGG using CGGA dataset. **c,d** Correlation between ssGSEA score of some states or processes and the CTL level in LGG using TCGA dataset (c) and CGGA dataset (d). **e** Logarithm of hazard ratios and inflammatory related signal pathways in LGG and GBM using TCGA (left) and CGGA (right).

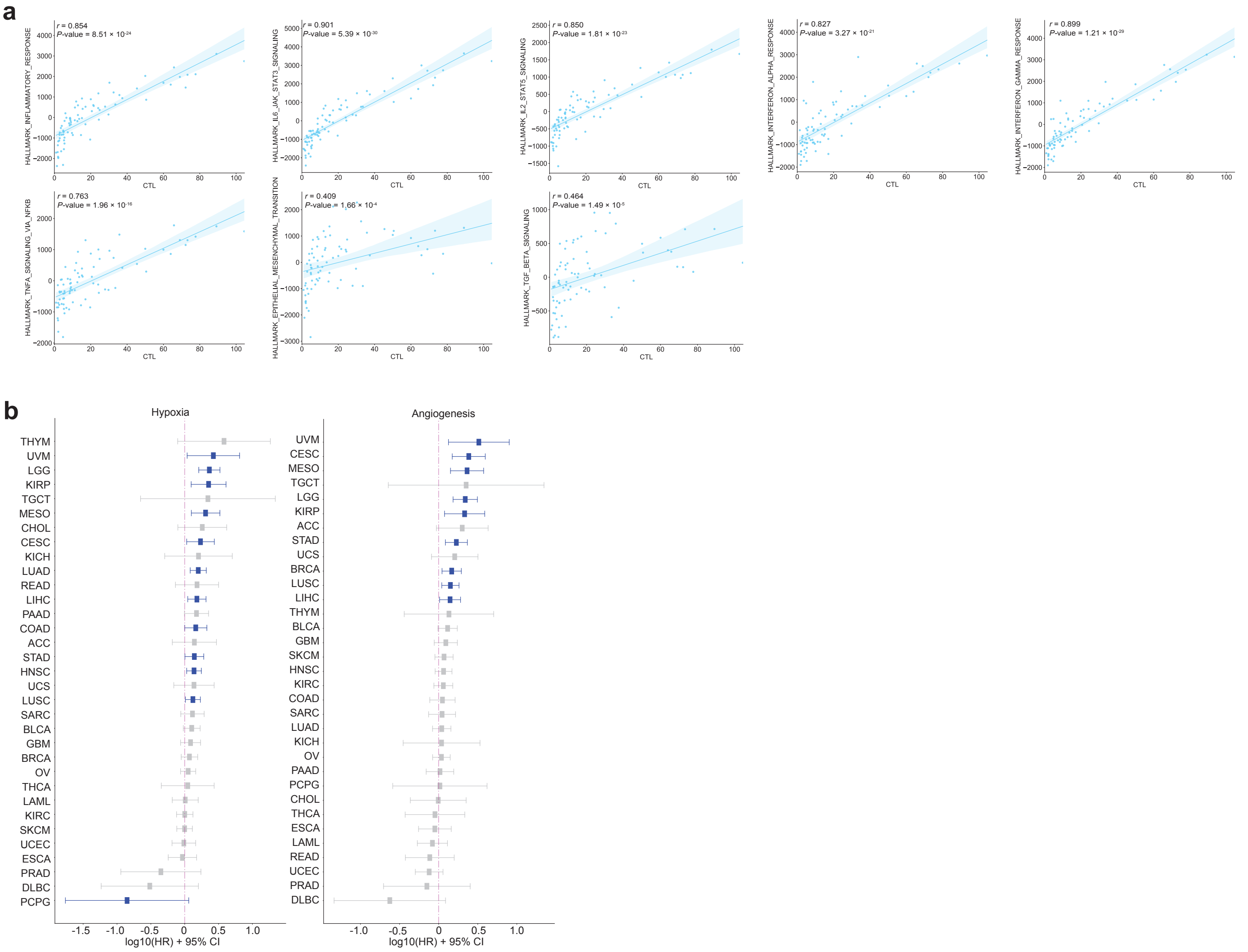

Figure S3 a Correlation between ssGSEA score of some states or processes and the CTL level in UVM. **b** Logarithm of hazard ratios for Hypoxia and Angiogenesis among cancer types. Those with $p<0.05$ are shown in blue and others are in gray.

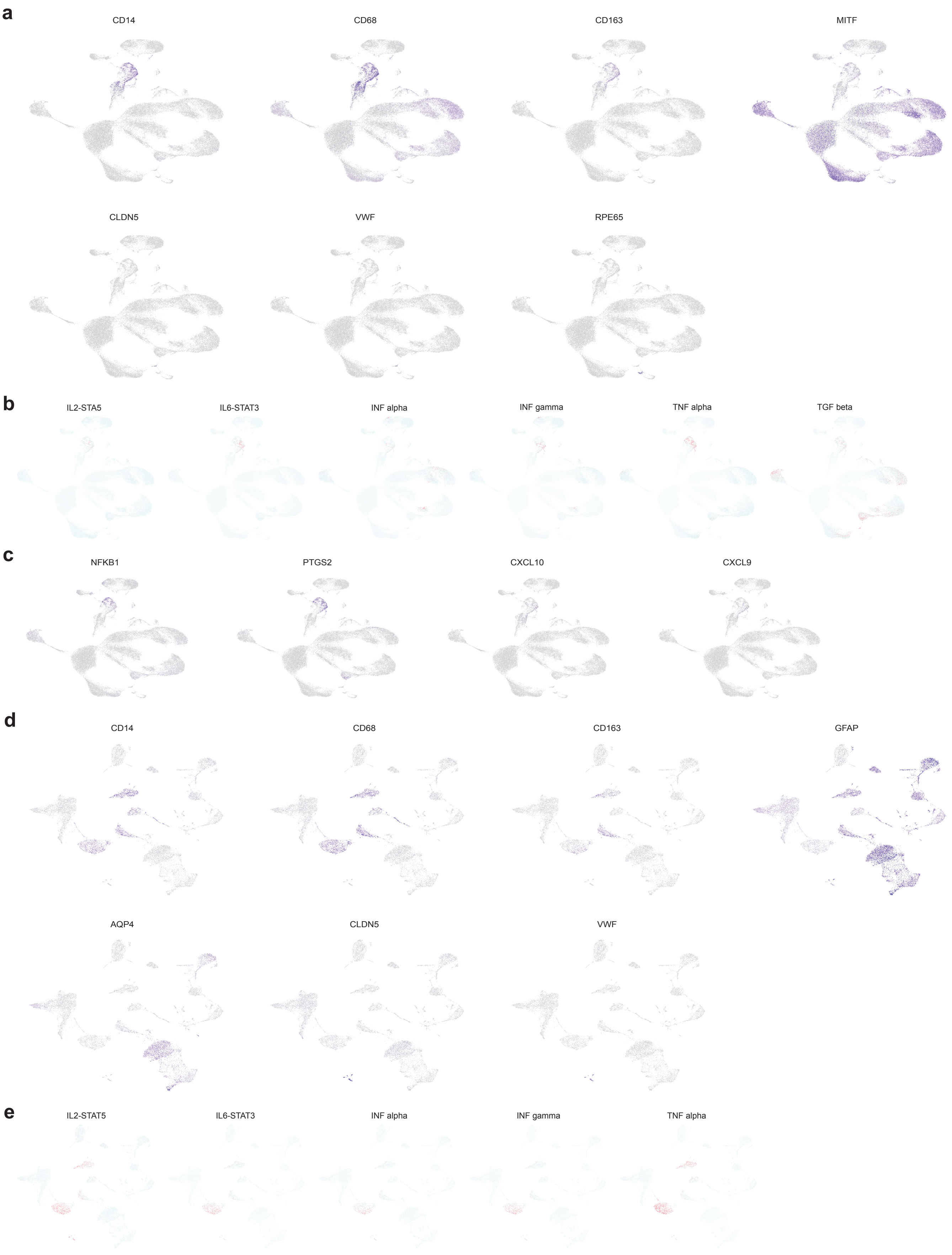
a
CD14
CD68
CD163
MITF
CLDN5
VWF
RPE65
b
IL2-STA5
IL6-STAT3
INF alpha
INF gamma
TNF alpha
TGF beta
c
NFKB1
PTGS2
CXCL10
CXCL9
d
CD14
CD68
CD163
GFAP
AQP4
CLDN5
VWF
e
IL2-STAT5
IL6-STAT3
INF alpha
INF gamma
TNF alpha

Figure S4 a,c,d Overview of marker gene expression for all cells in UVM (a, c) and LGG (d). **b,e** Overview of activation scores of some states and processes in UVM (b) and LGG (e).

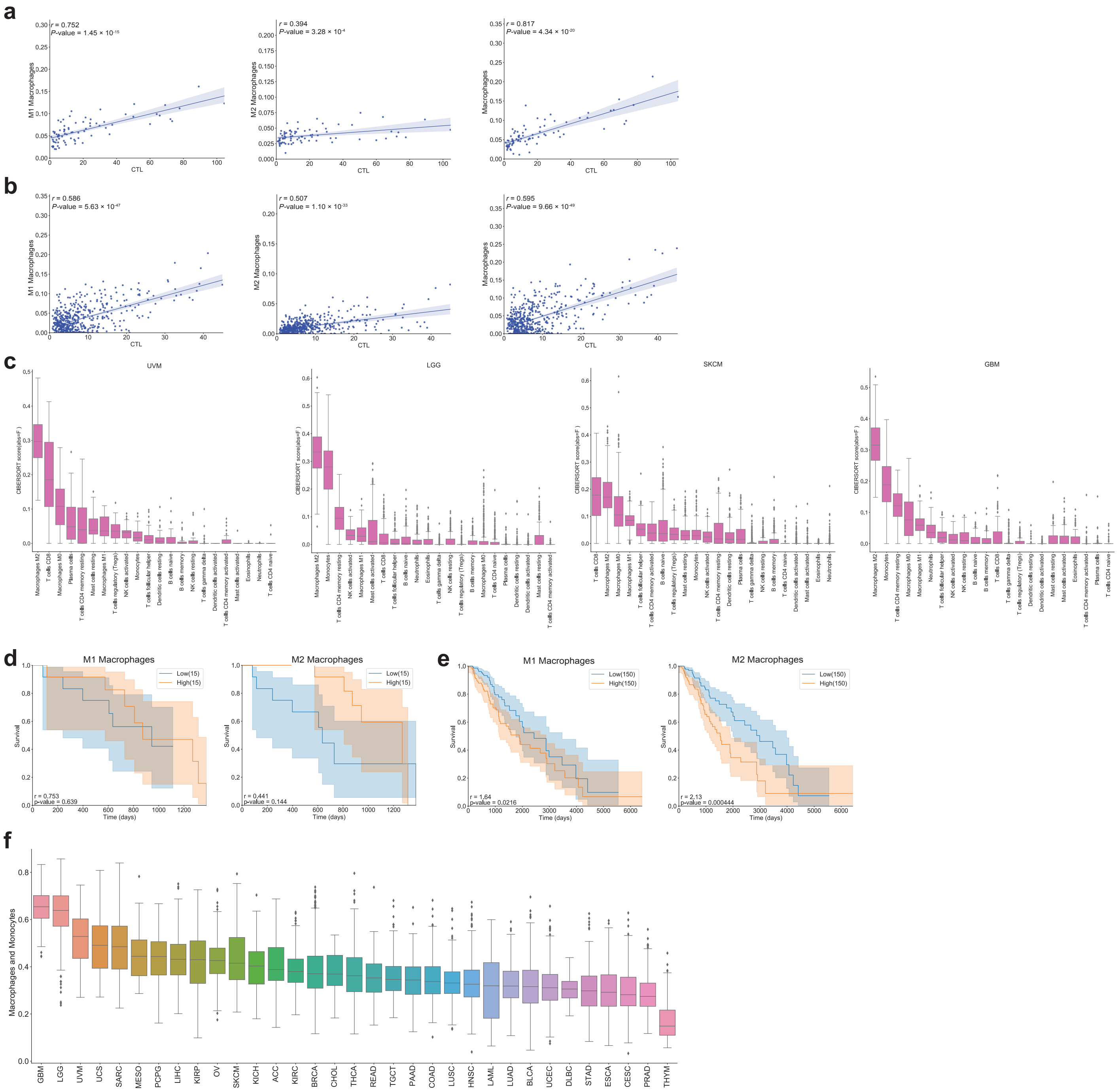

Figure S5 a,b Correlation between infiltration level of 'M1 Macrophage', 'M2 Macrophages' 'Macrophages' calculated by xCell and the CTL level in UVM (a) and LGG (b). **c** The fraction of each immune cell in tumor as determined by CIBERSORT (absolute=F) in UVM, LGG, SKCM, GBM. **d,e** Kaplan-Meier curves showing the association of M1and M2 macrophages with the prognosis in UVM (d) and LGG (e). **f** Percentage of macrophages ('Monocyte', 'M0 Macrophages', 'M1 Macrophages' and 'M2 Macrophages' combined) within the tumor across the cancer types as determined by CIBERSORT (absolute=F).
